# Microsatellite Organization in the Grasshopper *Abracris flavolineata* (Orthoptera: Acrididae) Revealed by FISH Mapping: Remarkable Spreading in the A and B Chromosomes

**DOI:** 10.1371/journal.pone.0097956

**Published:** 2014-05-28

**Authors:** Diogo Milani, Diogo Cavalcanti Cabral-de-Mello

**Affiliations:** UNESP - Univ Estadual Paulista, Instituto de Biociências/IB, Departamento de Biologia, Rio Claro, São Paulo, Brazil; Leibniz-Institute of Plant Genetics and Crop Plant Research (IPK), Germany

## Abstract

With the aim of acquiring deeper knowledge about repetitive DNAs chromosomal organization in grasshoppers, we used fluorescent *in situ* hybridization (FISH) to map the distribution of 16 microsatellite repeats, including mono-, di-, tri- and tetra-nucleotides, in the chromosomes of the species *Abracris flavolineata* (Acrididae), which harbors B chromosome. FISH revealed two main patterns: (i) exclusively scattered signals, and (ii) scattered and specific signals, forming evident blocks. The enrichment was observed in both euchromatic and heterochromatic areas and only the motif (C)_30_ was absent in heterochromatin. The A and B chromosomes were enriched with all the elements that were mapped, being observed in the B chromosome more distinctive blocks for (GA)_15_ and (GAG)_10_. For A complement distinctive blocks were noticed for (A)_30_, (CA)_15_, (CG)_15_, (GA)_15_, (CAC)_10_, (CAA)_10_, (CGG)_10_, (GAA)_10_, (GAC)_10_ and (GATA)_8_. These results revealed an intense spreading of microsatellites in the *A. flavolineata* genome that was independent of the A+T or G+C enrichment in the repeats. The data indicate that the microsatellites compose the B chromosome and could be involved in the evolution of this element in this species, although no specific relationship with any A chromosome was observed to discuss about its origin. The systematic analysis presented here contributes to the knowledge of repetitive DNA chromosomal organization among grasshoppers including the B chromosomes.

## Introduction

The accumulation of highly repetitive DNAs that are organized in tandem and dispersed is a common pattern in eukaryotic genomes [1]–[3]. Among tandem repeats, microsatellites or simple sequence repeats (SSRs) are composed of short motifs (∼6 bp) and constitute one of the most dynamic types of sequences; SSRs are abundant and can be located in specific chromosomal areas or widely scattered throughout euchromatic or heterochromatic areas [4]–[7]. In addition to other repetitive DNAs, such as transposable elements (TEs), satellite DNAs and multigene families, these sequences, have had a great impact on the organization and evolution of genomes [3], [5], [8]–[13].

One genome element that is characterized by the accumulation of repetitive DNAs are the B chromosomes (supernumerary or accessory chromosomes), which are dispensable elements that occur as polymorphism in addition to the standard karyotype in more than 2,000 eukaryotic species [14]–[16]. The close relationship between B chromosomes and repetitive DNAs has been demonstrated in some studies, revealing distinct classes of this genomic fraction, including transposons, retrotransposons, satellite DNA and multigene families. The accumulation of these repetitive DNAs may have resulted from a lack of recombination and may have led to B chromosome species-specific evolution [14]–[20].

The occurrence of B chromosomes has been reported in 191 Orthoptera species and in approximately 14.6% of Acridoidea representatives; B chromosomes also prevail in species with acrocentric chromosomes [21]. Although prevalent in grasshoppers, the molecular content of B chromosomes and their relationship with A elements have only been extensively investigated by the chromosomal mapping of repetitive DNAs in a few species, mainly in *Eyprepocnemis plorans* and to a lesser extent in *Locusta migratoria*, *Abracris flavolineata*, *Rhammatocerus brasiliensis*, *Xyleus discoideus angulatus*, *Dichroplus pratensis*, and species of *Podisma*. In these species, the mapping of repetitive DNAs has revealed variability and the presence of distinct sequences in B chromosomes, such as satellite DNA, rDNAs, histone genes, U2 snDNA and a SCAR marker; these mapping efforts may inform hypotheses about the possible origins and evolution of these elements [22]–[37].

Although a significant fraction of the eukaryotic genome is composed of microsatellites, their chromosomal distribution has been addressed only in specific groups (see for example [6], [7], [38]–[44]). Moreover, the studies that explore the composition of the B chromosome with distinct repetitive sequences do not systematically investigate microsatellite occurrence/accumulation [38], [45].

Grasshoppers possess large genomes and it could be directly related to the proliferation of repetitive DNAs, as recently demonstrated by genome sequencing of *Locusta migratoria* [46], that revealed ∼60% of repetitive DNAs. On the other hand, the organization of specific repetitive sequences, like microsatellites, are poorly know in this insect group, with chromosomal mapping of (AG)_10_ and (AC)_10_ restrict to *E. plorans* and *Chorthippus* sp, respectively [38]. Here in order to obtain a deeper knowledge of repetitive DNAs chromosomal organization for standard complement and B chromosome composition/evolution in grasshoppers we mapped 16 distinct microsatellite motifs in the chromosomes of *Abracris flavolineata* (Acrididae: Ommatolampidinae), a species presenting 2n = 23,X0 (male) and 2n = 24,XX (female) and with occurrence of B chromosomes [37]. Our results revealed intense spreading of distinct motifs in A complement and B chromosome.

## Materials and Methods

Cells bearing one B chromosome were obtained from embryos males (2n = 23,X0) or females (2n = 24,XX) following the protocol proposed by Webb et al. [47], with slight modifications. These cells were used in C-banding experiments according to Sumner [48] and in FISH experiments using microsatellites. At least 15 metaphase spreads were analyzed to describe the patterns of microsatellite distribution.

Specific microsatellites were labeled directly with Biotin during synthesis at the 5′ end and were used as probes (Sigma, St. Louis, MO, USA). The microsatellites included mono-, di- tri- and tetra-nucleotides: (C)_30_, (A)_30_, (TA)_15_, (CG)_15_, (CA)_15_, (GA)_15_, (TAA)_10_, (TAC)_10_, (GAG)_10_, (GAA)_10_, (GAC)_10_, (CAA)_10_, (CAC)_10_, (CGG)_10_, (GACA)_4_, and (GATA)_8_. The FISH experiments were performed with at least 300 ng of DNA according to the protocol proposed by Pinkel et al. [49] and with modifications reported by Cabral-de-Mello et al. [50]. The probes were detected using Streptavidin, Alexa Fluor 488 conjugate (Invitrogen, San Diego, CA, USA), and all preparations were counterstained with DAPI (4′, 6-diamidino-2-phenylindone) and mounted in Vectashield (Vector, Burlingame, CA, USA). The results were observed using an Olympus microscope BX61 that was equipped with a fluorescence lamp and the appropriate filters. Images were photographed using a DP70 cooled digital camera in grayscale, and the images were pseudocolored and posteriorly combined and optimized for brightness and contrast with Adobe Photoshop CS6.

## Results

**Figure 1:**
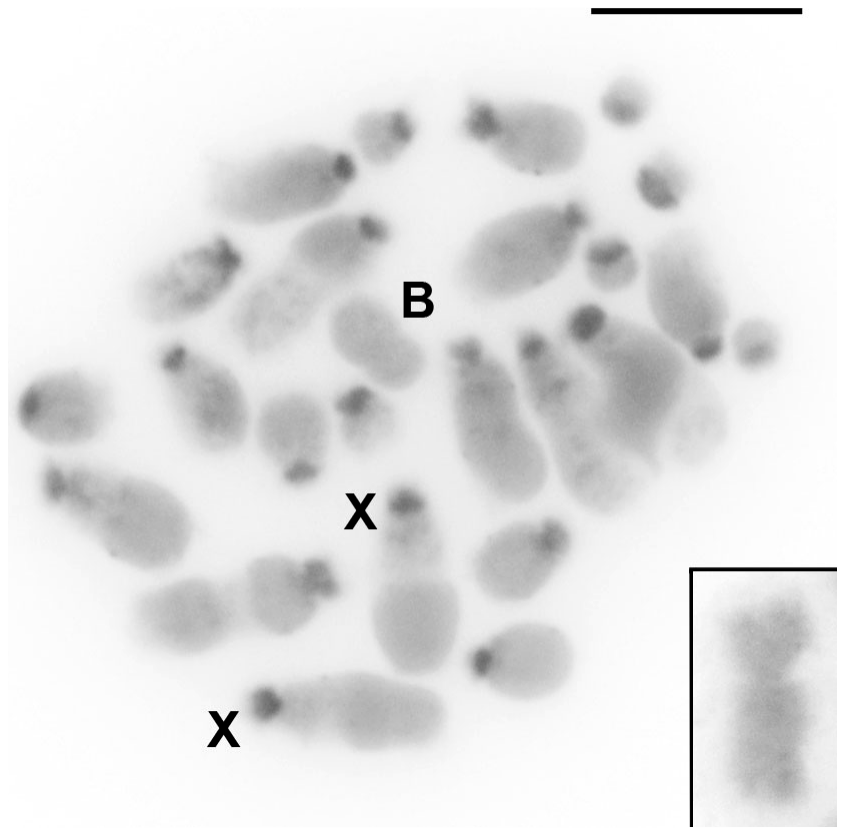
C-banding in embryo female mitotic metaphase of *Abracris flavolineata*. The X and B chromosomes are indicated. Note that the C-positive blocks in the centromeric regions extend to the short arms of the A chromosomes and the euchromatic nature of the B element. The inset highlights the euchromatic B chromosome. Bar  = 5 µm.

C-positive blocks that correspond to constitutive heterochromatin were observed in pericentromeric regions that extended to the short arms in the A complement. The B chromosome was euchromatic, as previously described by Bueno et al. [37] (Figure 1).

**Figure 2:**
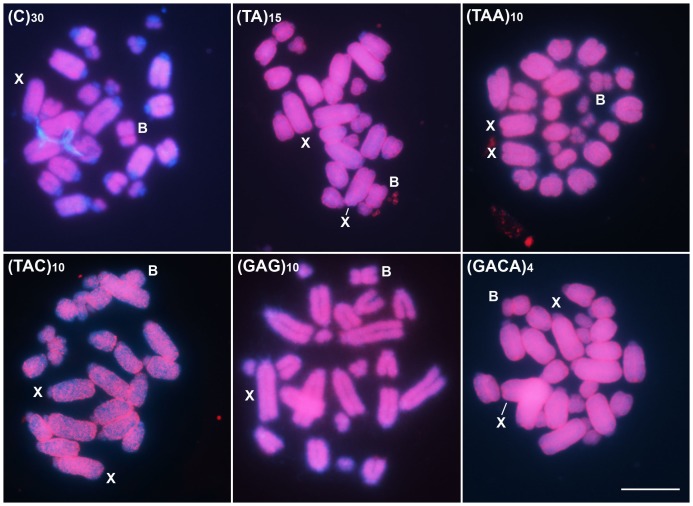
FISH mapping for six microsatellite motifs in embryo mitotic cells of *A. flavolineata* with scattered distribution. Each microsatellite is indicated in each image. The B and X chromosomes are pointed in each image and the sex of the embryo can be noticed by the occurrence of one (male X0) or two (female XX) X chromosomes. Note the absence of signals in the heterochromatic areas for (C)_30_. Bar  = 5 µm.

**Figure 3:**
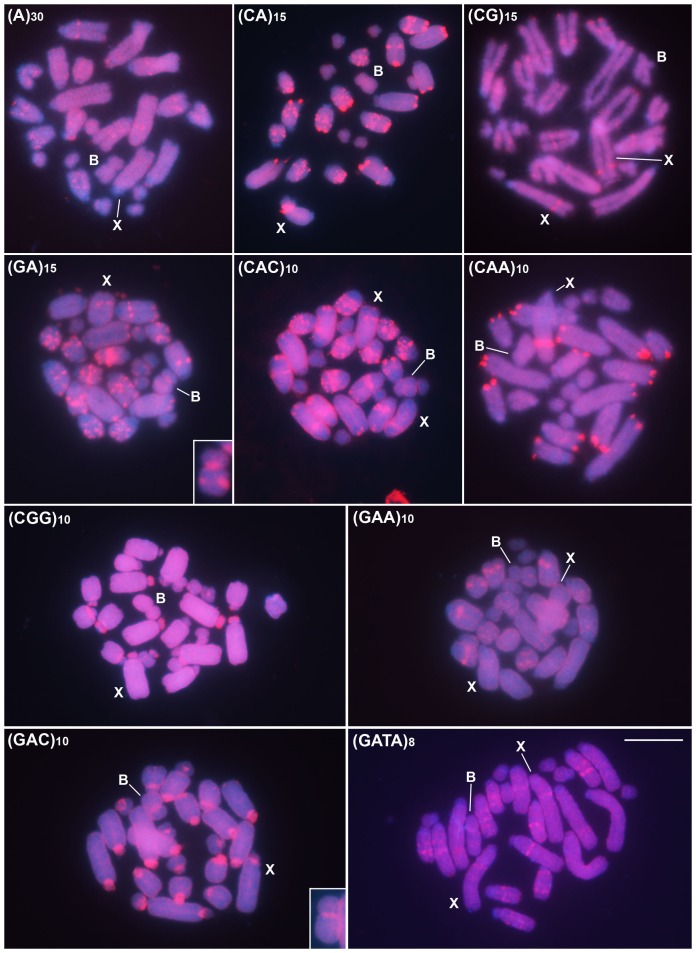
FISH performed using ten microsatellites as probes in embryo mitotic cells of *A. flavolineata* revealing distribution of repeats in distinct chromosomal regions. Each microsatellite is indicated in each image. The B and X chromosomes are pointed in each image and the sex of the embryo can be noticed by the occurrence of one (male X0) or two (female XX) X chromosomes. The insets highlight the B chromosome with probes for repeats with distinctive blocks. Bar  = 5 µm.

Two distinct general distribution patterns that depended on the microsatellite were observed by FISH: (i) exclusively scattered signals (Figure 2), and (ii) scattered and specific signals, forming evident blocks (Figure 3). Among the microsatellites with scattered distribution slight differences were observed for distribution with signals along the entire chromosomes for (TA)_15_, (TAA)_10_, (TAC)_10_, (GAG)_10_ and (GACA)_4_ and restrict to euchromatin for (C)_30_. Moreover the intensity of signals were distinct, being observed less intensity for (TAC)_10_ in comparison to the other repeats (Figure 2). Regarding the microsatellites with scattered and specific signals some differences were also remarkable for the evident blocks, as follows: (i) only interstitial signals were observed for (A)_30_ and (GAA)_10_; (ii) interstitial and terminal blocks were noticed for (CG)_15_, (CAA)_10_ and (CAC)_10_; (iii) interstitial, terminal and proximal blocks were noticed for (CA)_15_, (GA)_15_, (GATA)_8_; (iv) mainly blocks in the short arms were evidenced for (CGG)_10_; and finally (v) centromeric blocks, in some chromosomes extending to the short arms were observed for (GAC)_10_ (Figure 3).

Concerning the B chromosome, in general the microsatellites were scattered along its entire extension. More evident blocks were observed only for (GA)_15_, located in the centromeric and in the interstitial region of the long arm and for (GAC)_10_, with centromeric block (Figure 3). The X chromosome showed more intense interstitial hybridization for (CA)_15_, (CG)_15_, (GA)_15_, (CAC)_10_ and (CAA)_10_. For (CGG)_10_ and (GAC)_10_ more intense signal were noted in the short arm and centromeric positions, respectively (Figure 3).

## Discussion

This study provides the first systematic analysis for microsatellites repeats chromosomal mapping in a grasshopper species. On a broad scope, the results indicated that an intense spreading of mono-, di-, tri- and some tetra-nucleotides has occurred in the *A. flavolineata* genome, including similarly heterochromatic and euchromatic areas of the A complement and the B chromosome. The accumulation of microsatellites in eukaryotic genomes, in general, tends to vary and decrease from vertebrates and plants to fungi and invertebrates. It has also been suggested that the presence of highly repetitive simple sequences can correlate to genome size and in species distantly related the microsatellite quantity and genome size present correlation, as for example human, *Drosophila melanogaster* and *Saccharomyces cerevisae* and *Arabidospsis thaliana* [3], [51]–[54]. For grasshoppers, a survey performed in *Chorthippus biguttulus* (Acrididae) with expected large genome revealed that the microsatellites are not more frequent, at least the di-nucleotides studied, than in other insect with smaller genomes. On the other hand, the repeat arrays are longer than in other insects and could reflect the genome size increasing [54]. Non-correlation of genome size and abundance of microsatellite was also documented in other insects, as in parasitic wasps that have more microsatellite density than *D. melanogaster*, although their genomes are comparable in size [55]–[57].

A remarkable distribution/spreading of microsatellites, such as that observed for most of the repeats described herein, has also been reported in other species for distinct motifs, such as in reptiles (*Eremias velox*), plants (*Solanum licopersicum*, *Silene latifolia* and *Rumex acetosa*) and insects (*Drosophila melanogaster*), including the grasshopper species *Eyprepocnemis plorans* [38], [39], [41], [42], [58], [59]. This wide spreading could be attributed to the activity of transposable elements that contain microsatellite sequences and, in some cases, are involved with microsatellites origin during genome integration of TEs [8], [60]–[64]. In the case of *A. flavolineata*, the occurrence/spreading of some microsatellites in euchromatin resembles the distribution of two isolated *Mariner*-like transposable elements [65].

In contrast to intense and random distribution patterns, localized intense signals were observed for some microsatellites in *A. flavolineata*. Specific signals were observed in the chromosomes of the grasshopper *Chorthippus* sp for AC motif, and cases of non-random distribution for distinct repeats are well documented within and between *Drosophila melanogaster* chromosomes and in Triticeae plants [7], [38]; these patterns depended on euchromatin, heterochromatin and centromeric areas. Moreover, in some other cases, the density of SSRs varied between chromosomes in the same genome, as observed for *A. flavolineata*. These results indicate that the organization of SSRs could form a particular pattern for each repeat, which could follow distinct trajectories of expansion, elimination and accumulation at intra- and inter-genomic levels through distinct molecular mechanisms, such as ectopic recombination, slippage replication and transposition [40], [44], [66]–[69].

Concerning the B chromosome, which has been poorly investigated in the context of microsatellite mapping, the (CAA)_10_ repeat was reported to harbor these elements in rye (*Secale cereale*) [45], while the B chromosome of *E. plorans* did not revealed signals for (AG)_10_, that is abundant in the A chromosomes of the species [38]. As, in general, a non-recombining element with A elements the B chromosomes could be a preferable site for SSR accumulation during its evolution; SSR accumulation may be involved in the differentiation process of these chromosomes, which would favor, for example, the rate of mutability. The mutability rate for microsatellites is very high (10^−2^ to 10^−6^ events per locus per generation) in relation to those at coding gene loci [8]; this high mutability could have been occurred in the A complement and B chromosome of *A. flavolineata*, which exhibit similar enrichment of most microsatellites that have been mapped here. In contrast, there is apparently a paucity of general pool of repetitive DNAs in the B chromosome of *A. flavolineata*, which was determined by using the *C_0_t*-1 DNA fraction (composed of highly and moderately repetitive DNAs) as a probe [37], indicating that not all types of repetitive DNAs have been isolated in this fraction, such as distinct microsatellites. The distinctive block for (GA)_15_ observed in the long arm of the B chromosome in *A. flavolineata* indicate that after its origin through isochromosome formation [37] the arms probably have been experienced distinct accumulation of repetitive sequences, as also recently reported for a *Mariner*-like transposable element (*Afmar2*) [65].

Although we noted the spreading of SSRs in the non-recombining B chromosome it was, in general, not different from A chromosomes that present distinct degrees of recombination, e.g., recombining (autosomes) and less-recombining (X chromosome that recombine only in females). Furthermore, these differences, in general, were not observed for distinct chromosomal regions with distinct degree of recombination, such euchromatin and heterochromatin. Such differences of microsatellite accumulation between different chromosomes with distinct degrees of recombination have been previously reported. For example, an accumulation of distinct microsatellites was observed in the young Y chromosome of *Rumex acetosa* [42]. Additionally, *Silene latifolia*, which has non-recombining genome regions, e.g., the Y chromosome, has also accumulated microsatellites in this chromosome [39]. The accumulation of microsatellites in sex chromosomes has also been reported in animals, such as for example in *Eremias velox* (lacertid lizard) [41], *Aprasia parapulchella* (Pygopodid lizard) [43] and *Semaprochilodus* (Prochilodontid fish) [44], suggesting a role for this type of sequence in sex chromosome differentiation favored by the suppression of recombination among these elements.

Although the results of this study did not provide new insights regarding the specific origin of the B chromosome in *A. flavolineata*, proposed to be derived from pair one [37], our approach provided novel valuable primary data about the organization of distinct microsatellite sequences in grasshopper genomes, including the A complement and B chromosomes. Moreover, these data increase the knowledge of B chromosome composition in this insect group and in eukaryotes, indicating that microsatellites could play important role in B chromosome evolution. The next step should employ the use of this simple assay, i.e., FISH mapping of microsatellites, in other grasshopper species that bear B chromosomes to determine whether the spreading of these elements commonly occurs in this group and whether there is a correlation with the distribution of euchromatin/heterochromatin.
